# Leadership and crisis management and their link to improvement of hotel performance: A study of the Jordanian hotel sector

**DOI:** 10.1016/j.heliyon.2023.e17839

**Published:** 2023-07-02

**Authors:** Amar Hisham Jaaffar, Raed Hussam Alzoubi, Omar Hamdan Mohammad Alkharabsheh, Jegatheesan Rajadurai

**Affiliations:** a Institute of Energy Policy and Research (IEPRe), Universiti Tenaga Nasional, Malaysia; b Department of Administrative Sciences/Crisis Management, Al-Balqa’ Applied University, Jordan; c Department of International Business, Faculty of Accountancy and Management, Universiti Tunku Abdul Rahman (UTAR), Malaysia; d UNITEN Business School, Universiti Tenaga Nasional, Malaysia

**Keywords:** Crisis management, Hotel performance, Jordan, Leader’s experience, Leadership styles

## Abstract

The current environment of volatility, uncertainty, complexity and ambiguity has created a prolonged state of uncertainty for the Jordanian hotel industry. Crisis management leadership is one of the most important attributes for a hotel. The main aim of this study is to evaluate the mediating role of crisis management, the moderating role of a leader’s experience, their relationship to styles of leadership (transformational and transactional) and the resultant performance of Jordanian hotels. Research was based on a self-distributed questionnaire survey of 119 respondents currently holding managerial positions in Jordanian 3 to 5 star hotels. Partial Least Square Structural Equation Modelling was then employed. The findings suggest a transformational leadership style and crisis management experience are the most important attributes for a leader to sustain hotel performance during a crisis. Leaders with a transactional leadership style need crisis management skills to sustain hotel performance rather than experience which is not as important in their case. This paper proves that different leadership styles have a different influence on a hotel’s survivability during a crisis. Therefore, a hotel’s management group must ensure that a leader with an appropriate leadership style takes control during these situations. By combining leadership attributes, experience, and crisis management in a comprehensive framework to ensure sustainable hotel performance in the face of a crisis, this study adds to the body of knowledge on leadership and crisis management practices.

## 1 Introduction

Political challenges remain in the Arab region, especially following the Arab Spring and the on-going Arab-Israeli dispute which has taken a toll on the Arab and Jordanian tourism industry. Furthermore, despite Jordan’s many attractions, Jordan has been unable to make use of its touristic potential due to the large number of crises, particularly the fallout from terrorist attacks that have adversely affected its tourism industry [1]. According to Altarawneh et al. [2], the Middle East is reputed to be a challenging region because any event that takes place in any part of the region has a ripple-on effect for all countries within the region. For Middle Eastern countries, the security situation negatively impacts the number of visitors travelling to the Middle East [3]. As a consequence, the potential for a crisis to erupt in the Middle East, as well as negative stereotypes, has prompted Middle Eastern countries to concentrate on developing crisis management strategies and to focus on countering the negative stereotypes [4]. However, in 2011 and 2012, Jordan, along with other countries in the Middle Eastern region, were shaken by both violent and non-violent protests known as the Arab Spring [2], as well as the emergence of militant terrorist groups such as the Islamic State of Iraq and Syria [5].

In Jordan, the hotel sector was the major player in the tourism industry, accounting for around 30% of the workforce and nearly 25% of tourism revenue [6]. As a result of the unrest, the hotel sector was badly hit, resulting in a fall in hotel revenue, hotel jobs and the viability of the whole industry [6,7]. Furthermore, as the hotel industry in Jordan suffered from a recession and low occupancy rates, many tourist establishments were forced to borrow from local banks to meet their obligations and accumulated debts [8]. All of these factors contributed to the poor performance of hotels, which were unable to operate since their revenue could not cover their operating costs, which in turn affected the calibre of their hospitality services. This underscores the importance of managing crisis events with effective and efficient leadership that possesses the ability to apply and implement crisis management strategies at all phases of the crisis.

According to an annual report issued by the Jordan Hotel Association [8], there was huge expenditure on the part of hotels, such as the installation of luggage inspection devices, cameras and security arrangements, to satisfy government requirements. All these measures imposed additional financial burdens and costs on the hotel sector. However, despite the fact that hotel revenues were falling, hotels still had to pay large on-going taxes to the government. These taxes represent an annual burden for hotels and make it even more difficult for them to make a profit. These issues, and those highlighted earlier, provided the motivation for this present study.

The tourism industry is very vulnerable to crises. For example, the COVID-19 pandemic is one of the most recent critical problems for Jordan’s tourism sector. The pandemic has been deemed a global public health crisis and has led to many restrictions on movement and travel. This has had a serious effect on the hotel sector in Jordan which is experiencing a drastic fall in business. The pandemic, combined with terrorist attacks and major socio-political disturbances in the Middle East, have had a truly damaging effect on the tourist industry. In Jordan, a significant and steady rise in the number of tourist arrivals was experienced from 2005 to 2010, but the impact of the Middle East crisis, which worsened at the start of 2010, resulted in a significant drop in tourist numbers from 8 million in 2010 to 5 million in 2019 [9]. Furthermore, additional data reveals a 41% drop in 2017 with anecdotal evidence suggesting further falls in tourist arrivals in 2018 [2].

The decline in tourist numbers has led to the low occupancy of hotel rooms and a significant loss of hotel stock and revenue [10], resulting in a negative impact on the hotel sector and posing a challenge for hotel performance [6,11]. The vulnerability of this sector in the face of crisis situations is higher than in other economic sectors because of the special features of the hotel business and, therefore, the notion and awareness of crisis management must play an essential role in the operations of hotel companies. Where various forms of crises have been of major concern to the tourism and hotel industry, the degree of crisis management expertise on the part of hotel managers is of paramount importance [12]. As stated by Fener & Cevik [13], organisational leaders should be knowledgeable and skilled so they can effectively overcome sudden states of crisis. If organisations are not managed well during crises, new problems will inevitably arise. However, the relationship between leadership styles and organisational performance is inconclusive and needs to be further investigated [14].

Bowers, Hall & Srinivasan [15], claim that, for many organisations in crisis, their ability to survive depends on the individual in charge at the time. Furthermore, considering the importance of the hotel sector to the Jordanian economy in terms of providing income and job opportunities, hotel performance during crises is an area ripe for research efforts to ensure the viability of the Jordanian hotel industry. This study chose the transformational and transactional leadership styles because prior research in the context of the Middle East hotel industry demonstrated that a leader’s approach and techniques of leadership can lean toward either the transactional or transformational [16,17]. Furthermore, a leaders’s behaviour and mindset are controllable variables which are critical in preventing them from overreacting to yesterday’s events, such as a crisis, and allowing them to look ahead to improve their firm’s performance. This study proposed and tested a conceptual model to investigate the impact of Jordanian hotel leaders' leadership styles and experiences, as well as crisis management practices, on hotel performance during a crisis. The model was also used to test the moderating effect of leaders' experience and the mediating effect of crisis management practices. Transformational leadership is the extent to which a leader’s behaviour/attitudes can increase their team’s abilities, aspirations and motivation to sacrifice their self-interests for the organisation’s goals [18]. Transactional leadership involves leaders using rewards and promises of rewards to improve their teams''/employees' performance [19]. Crisis management is an ongoing integrated and comprehensive effort that organisations effectively implement to firstly understand and prevent a crisis, and then effectively manage those that occur, taking stakeholders' interests into account at every step of their planning and training process [20]. A leader’s experience is comprised of their number of working years and their social capital applied to their current job and workplace [21]. Hotel performance is the result of hotel operations. To assess hotel performance, its four dimensions—financial, customer, internal process, and learning and growth — must be combined and viewed as a whole [22]. The questionnaire was developed using scales recognised in the literature on aspects such as leadership styles and experience, crisis management, and hotel performance. The reliability and validity of all constructs showed that the questionnaire was appropriate for measurement. The questionnaires were self-distributed to 148 owners and senior management of three-to five-star hotels in Jordan. 119 accepted questionnaires data were analyzed using Partial Least Square (PLS) Structural Equation Modelling (SEM). This study contributes to the knowledge of leadership and crisis management during a prolonged crisis period focusing on the hotel industry in a volatile region like Jordan. It also utilized single-framework by incorporating the main leadership styles (transactional and transformational leadership styles) as an independent variable, crisis management as a mediating variable, leader’s experience as a moderating variable, and hotel performance as a dependent variable. It also demonstrates the importance of an appropriate leadership style possessed by hotel top management to devise crisis management strategies that subsequently contribute to the hotel’s performance. Timely recommendations for future studies, the hotel industry, and governments are provided to cope with the crisis.

## 2 Literature review

### 2.1 Transformational and transactional leadership styles and hotel performance

Early studies and analysis have shown that organisational performance and transformational leadership are significantly related. Spitzbart [23], found that the effect of transformational leadership and transactional leadership on employee satisfaction was important in a hotel management performance study. In a study of performance in the hospitality industry in Thailand, Zumitzavan & Udchachone [24] reveal that there is a positive relationship between transformational leadership and successful performance. Hashim et al. [25] found that transactional and transformational leadership styles had a significant correlation with the performance of organisations comprising Malaysian small and medium-sized enterprises. Furthermore, Cheung, King & Wong [26] argue that a transformational style of leadership greatly influences hotel performance. Sobaih et al. [17] discovered in their study of employees in Egypt’s deluxe hotels that transformational leadership has a positive effect on organisational commitment and employee intent to remain in the company. From the point of view of the Contingency Theory, the success of transformational leadership depends on the situation the firm is in at the time of the crisis [27]. According to the Resource Based View, transformational leaders can be viewed as intangible resources in increasing hotel performance, such as during a crisis [28].

The following hypothesis for research is therefore proposed:

H1 *The transformational leadership style has a positive relationship with hotel performance in the Jordanian hotel sector*.

Howell & Hall-Merenda [29] assert that a transactional leadership style has a positive effect on an organisation’s performance, while Nazarian, Soares & Lottermoser [30] report that there is evidence in literature that indicates that transactional leadership is an important element in an organisation’s performance assessment. A transactional style of leadership enables organisations to attain their current goals by relating performance to rewards and provides the necessary resources needed for workers to perform a task [31].

Elenkov [31] studied the influence of leadership on an organisation’s performance in Russia and found that managers engaging in transactional leadership had a significant impact on the organisation’s performance and innovation. A study by Radwan [32] reveals that transactional leadership is the dominant style used in four-star hotels in Egypt but had an adverse effect on employees' performance, job satisfaction and motivation. However, it found that transformational leadership is strongly associated with employees' creativity and performance.

Chiang & Wang [33] state that the dimensions of the transactional style of leadership had a positive effect on organisational commitment and trust in the hotel sector. Aziz et al. [34] observe that the relationship between the transactional style of leadership and organisational performance was significantly positive in their study of small and medium-sized enterprises in the service industry. It was also observed within the Malaysian service industry that transactional leadership had a positive effect on the outcomes of organisations [35]. Alzoubi and Jaaffar [16], on the other hand, believe that transactional leadership has no significant relationship with hotel performance in the context of the Jordanian hotel industry. From the perspective of the Contingency Theory, the transactional style of leadership is better at preventing a drop in firm performance during a crisis [36]. According to the Resource Based View, transactional leadership aspires to strengthen the firm’s culture, strategy and structure, which can improve firm performance during a crisis [37].

The following hypothesis for research is therefore proposed:

H2 *The transactional leadership style has a positive relationship with hotel performance in the Jordanian hotel sector.*

### 2.2 Crisis management and hotel performance

Crises can adversely affect any organisation [38]. Crisis management was identified in a recent study as one of the key success drivers in the hotel industry [12,16,39]. However, despite the frequency and the negative impact of crises, the study of crisis management in the tourism industry has received only limited attention, thus creating the need for more research into this issue [39]. Furthermore, Al-Omari et al. [6] argue that the crisis that occurred in the Arab region as a result of the Arab Spring in 2011 had a negative influence on hotel performance. This strengthened the need for the creation of crisis management strategies, as this gives scope for innovation in the hotel sector. Majli & Tamimi [40] examined the influence of a crisis management approach on employees' performance in the manufacturing sector of Jordan. Analysis revealed that crisis management significantly impacted workers' performance. The findings of Yu, Stafford & Armoo [41] reveal a positive correlation between crisis management and hotel performance and propose that crisis management ought to include crisis preparation, crisis containment and crisis recovery. In addition, in his empirical research, Labaš [42] noticed that there was a statistically significant positive effect associated with crisis management (crisis preparedness) on organisational performance. These findings are supported by the Contingency Theory which posits that during a crisis, the performance of organisations is contingent on quick responses to crisis situations and the engagement of stakeholders [43]. The following hypothesis for research is therefore proposed:

H3 *Crisis management has a positive relationship with hotel performance in the Jordanian hotel sector.*

### 2.3 Transformational and transactional leadership styles and crisis management

Previous studies such as those by Burns [44] and Zhang, Jia & Gu [45] argue that in crisis situations, transformational leaders, as well as their followers, provide mutual help in advancing to a higher crisis management level. Moreover, Tracey & Hinkin [46] assert that hospitality managers and executives encounter critical and difficult situations which require high levels of knowledge and ability. Mumford et al. [47] state that transformational leaders direct staff members through the process of clarification and comprehension during crisis situations by articulating a convincing vision which highlights the importance of going beyond personal interests to work for the general interests of the team; this will invariably lead to the organisation’s survival. Cho & Tseng [48] noticed that transformational leadership had been adopted by managers in the financial crisis. Zhang et al. [45] propose that transformational leaders can inspire their workers to accomplish tasks in a crisis situation more efficiently and effectively, by thinking clearly and displaying self-sacrifice. Researchers [[49], [50], [51]] evaluated the role played by leadership styles during crisis management and found that a leadership style such as transformational leadership has a positive effect on crisis management. Therefore, the following hypothesis for research is proposed:

H4 *The transformational leadership style has a positive relationship with crisis management in the Jordanian hotel sector.*

Transactional leadership employs monitoring, organisation and performance management practices to improve employee performance through the use of both incentives and sanctions. It supports the preservation and maintenance of processes and practices for crisis management created by top management. Consequently, the findings of Hasan & Rjoub [52] and Alkhawlani [53] show that transactional leadership directly affects organisational crisis management. Their findings revealed that transactional leadership is an effective method for strengthening employee loyalty in support of organisational objectives because it provides followers with clarity on how to achieve the objectives and the rewards (both extrinsic and intrinsic) that they obtain from such achievements. This stimulates them to greatly improve their levels of productivity [54]. Leaders who utilise transactional leadership actions help their organisations recover from a crisis situation by showcasing a complex set of qualities in all five stages of crisis management (signal detection, preparation and prevention, containment, recovery and learning [55]. Furthermore, Kapucu & Ustun [56] found that core transactional leadership qualities positively influence crisis management effectiveness. In his study, Alkhawlani [53] also found that the transactional style of leadership exerts the most influence on crisis management.

Moreover, Heuvel [57] concludes that, in uncertain times and times of crisis, a transactional style of leadership has inherent qualities which assist the organisation to overcome the crisis situation. Zohar & Luria [58] also assert that a transactional style of leadership positively influences the outcomes during a crisis because it assists the organisation to implement very complex modes of operation. Therefore, the following hypothesis is proposed:

H5 *The transactional leadership style has a positive relationship with crisis management in the Jordanian hotel sector.*

### 2.4 Transformational leadership and transactional leadership style, crisis management and hotel performance

By proposing crisis management as the mediator, it will assist leaders to manage a crisis; this is because crisis management is a practice that takes into account both internal and external determinants that positively strengthen the company during the crisis. As a consequence, it can be argued that, in order for leaders with transformational or transactional leadership styles to improve their firm’s performance during a crisis, they need to have crisis management skills to help them manage situational factors [52]. This study found that the intervening role of crisis management strategies has a mediating effect which gives rise to the possibility of an indirect association between leadership styles and hotel performance; the mediator (crisis management) plays an intermediary role. This argument is supported by Zhao, Lynch Jr & Chen [59], as well as Nitzl, Roldan & Cepeda [60]. A direct relationship might also result from the presence of a significant mediator. Alkharabsheh, Ahmad & Kharabsheh [61] challenge the idea that transformational leaders effectively handle crisis situation more effectively than transactional leaders while proposing that future studies should focus on the interaction between these styles of leadership. Contingency Leadership Theory explains the rationale of crisis management actions during a crisis [43,62]. The three elements of Contingency Theory which emerge are: positional power, leader-member relationship and task-structure. They are used in several scenarios of crisis management and employ various skills of leadership [62]. This information is very useful in determining what type of leadership style is partially or fully mediated by crisis management leading to hotel performance. Along a similar line of enquiry, Yavuz & Zehir [63] found that crisis management had a mediating effect on the association between organisational learning and the performance of the firm. Nematova et al. [64] also observed the mediating role of crisis management on social media and software engineering collaboration tools and business performance. Similarly, the findings of the study of ALnuaimi et al. [65] support the mediating role of crisis management. When viewed through the lens of the Resource-Based View Theory, crisis management practices can be interpreted as well-structured strategies for gaining a competitive advantage in the midst of a crisis.

From these arguments, the following research hypotheses are put forward:

H6 *Crisis management has a mediating effect on the relationship between transformational leadership style and hotel performance in the Jordanian hotel sector.*

H7 *Crisis management has a mediating effect on the relationship between transactional leadership style and hotel performance in the Jordanian hotel sector.*

### 2.5 Transformational leadership and transactional leadership style, Leader’s experience, and hotel performance

Leadership experience contributes to better organisational performance [[66], [67], [68]]. Naqvi [69] reports that managerial experience is a vital factor in the success of SMEs in Pakistan. Furthermore, Al Saleh [70] detects a positive relationship between the experience of managers in previous work situations and the performance of an organisation. The results of a study by Alharbi, Yahya & Ahmed [71] reveal that appropriate levels of experience of managers positively affect the performance of SME’s. Similarly, Isaga [72] identifies experience as a vital determinant for a positive relationship with the performance of Tanzanian SMEs. Furthermore, Bonface, Malenya & Musiega [73] observe that there is a significant positive correlation between managerial experience and expertise and the performance of Kenyan investment banks. Consequently, leader’s experience is perceived to have a significant influence on hotel performance as the empirical findings demonstrate that organisations managed by experienced leaders fare and perform better than those not led by experienced managers. From these arguments, the following research hypotheses are put forward:

H8 A l*eader’s experience moderates the relationship between transformational leadership style and hotel performance in the Jordanian hotel sector.*

H9 A l*eader’s experience moderates the relationship between transactional leadership style and hotel performance in the Jordanian hotel sector.*

**Fig. 1:**
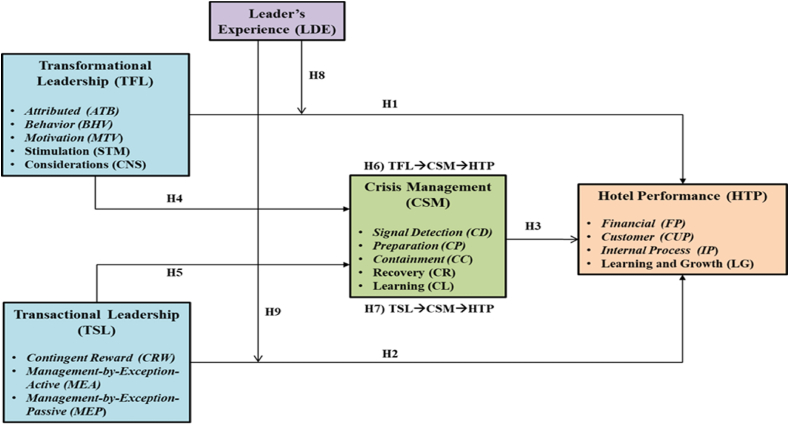
Theoretical framework and research hypotheses.

The integrated model framework proposed in this research is shown in Fig. 1.

## 3 Methodology

### 3.1 Sample and procedure

The current study was explanatory (causal) in research design, utilizing a self-administered survey questionnaire to implement a cross-sectional time horizon. The analysis unit consisted of hotels classified as three-star to five-star in Jordan. The survey was conducted in Jordan by hotel owners and senior management of hotels. The data was collected in the fourth quarter of 2019 when political volatility in a neighbouring country to Jordan reached its climax and the COVID-19 pandemic started to hit the world. According to the Jordan Hotel Association (JHA) list, 158 hotels met the sampling frame criteria; these included 84 three-star hotels, 38 four-star hotels, and 36 five-star hotels. The three-star and above hotels were chosen because they have larger organisational structure relative to the other hotel classifications, as well as being more likely to have formulated strategies to deal with crisis situations [74]. To facilitate judgmental sampling, the hotels included in the study (three-star to five-star) were chosen and the respondents canvassed. The surveys were distributed to the hotel senior management using a self-administered questionnaire for 146 hotels. 146 questionnaires were disseminated in total and 126 responses were received, making a response rate of 86.3%. Of the 126 questionnaires obtained, 7 were unsatisfactory, leaving 119 questionnaire surveys fit for examination. All participants in the study provided verbal informed consent, and they were informed that their anonymity and privacy would be strictly protected. Moreover, the Jordan Hotel Association (JHA) and the Ministry of Tourism and Antiquities (MOTA) Jordan both monitored and endorsed the ethical standards during the data collection process.

### 3.2 Measurement

The study used a survey questionnaire as the research instrument. Transformational leadership (TFL) was measured using a five-dimension framework of 20-items scale adopted from Avolio & Bass [75] and relating to leadership style in terms of idealized influence (attribute and behaviour), inspirational motivation, intellectual stimulation, and individualized considerations. Transactional leadership (TSL) was measured with three-dimensions of 12-items scaled adopted from Avolio & Bass [75] and relating to a leadership style which put emphasis on contingent rewards, and management by exception (active and passive). Alkhawlani et al.’s [76] 28-items scale was adopted to measure crisis management (CSM) comprising five phases of crisis management practice such as crisis signal detection, crisis preparation, crisis containment, crisis recovery and crisis learning. Hotel performance (HTP) was measured using four-dimensions of 17-items pertaining to scaled hotel performance adopted from Mohammed et al. [77]. It viewed hotel performance from four perspectives including finances, customers, internal processes, and learning and growth. All items were operationalized using a five-point Likert scale from 1 = “strongly disagree” to 5 = “strongly agree”. Back-translation was conducted to make sure that the Arabic version accurately captured the meaning in the English version of the questionnaire. The Arabic questionnaire was then pilot-tested on 30 hotel managers in Jordan who were then excluded from the primary study. The reliability calculated for the constructs in this research ranged between 0.764 and 0.929, all of which were higher than the cut-off point of 0.7 [78].

### 3.3 Demographic profile of respondents

The respondents differed in their demographic profiles which was very evident in the results of the sample profile’s descriptive analysis. The survey questionnaires were distributed in the mid, southern and northern regions of Jordan to hotels classified as 3-star, 4-star and 5-star.

Of the total number of respondents, 79.8% were male and 20.2% were female. 57.1% of the respondents were between the ages of 36–45 years. All of the respondents held managerial positions in administration, with most being in the category of department manager (71.4%). Therefore, for their respective hotels, all respondents were deemed suitable for answering the questions regarding hotel performance as well as crisis management. Furthermore, the respondents were considered to have adequate knowledge of crisis management phases as some of them (39.5%) had a working experience of not less than 14 years.

With respect to the hotel profile, 54.6% of the surveyed hotels were 3-star and most of their affiliations were independent (82.4%). The majority of the respondents (71.4%) came from the mid-region because this region has most of the 3-star to 5-star hotels in Jordan [8]. This was followed by the southern region (26.9%) and lastly the northern region (1.7%). 66.4% of the surveyed hotels had been in operation for more than 14 years thus they could be deemed as mature hotels. A considerable number of these hotels (33.6%) located in the mid-region had 51-100 rooms. Some of the hotels had a workforce of 50 employees or fewer (42.9%) - especially hotels in the 3-star category.

## 4 Results

### 4.1 Data analysis

The number of questionnaires that were valid was 119. Using SPSS 26.0, the descriptive statistics of each variable, including leadership attributes, crisis management, and hotel performance, were examined. To conduct a multiple regression analysis in complicated models like this study, partial least squares-structural equation modelling (PLS-SEM) is thought to be particularly effective for processing skewed data, exploratory research, or theoretical extensions [79]. In the data analytics of PLS-SEM as introduced by Henseler, Ringle & Sinkovics [80], a two-step method was used. The first step included the study of the measurement model, while the second step evaluated the structural relationships between the latent constructs. In order to evaluate the structural relationship of the model, a two-stage method was used to determine the reliability and validity of steps. F^2^, Q^2^, and R^2^ were calculated to evaluate the structural model. PLS-SEM analysis was conducted using SmartPLS version 3.3.7. A two-sided test with a significance level of 0.05 was used for all tests.

### 4.2 Descriptive analysis

The descriptive function was calculated by the covariance matrix procedure for all variables. The initial measurement item scores were parcelled so that the composite scores of all the variables could be calculated. A parcel is the sum or average of various individual items or indicators based on the factor loadings they are place on the construct [81]. Table 1 shows the mean and standard deviation of the models assessed using a Likert scale of 5 points:

**Table 1:** Results of descriptive statistics for variables.

Constructs	Mean	Standard Deviation	Minimum	Maximum
Transformational Leadership (TFL)	3.024	0.576	1.7	4.1
Transactional Leadership (TSL)	3.028	0.614	1.7	4.7
Crisis Management (CSM)	3.002	0.579	1.8	4.2
Hotel Performance (HTP)	3.004	0.481	1.7	4

As Table 1 shows, all mean values of the constructs (except crisis management) were higher than the mid-point value of 3. This was determined by the application of the mean as the measure of central tendency. This shows that the consensus of opinion of the respondents concerning the constructs is higher than the average. Transactional leadership has a mean value of 3.028 (highest mean value), while crisis management has a mean value of 3.002 (lowest mean value). The extent to which the individuals differed from the variable mean within each variable was indicated by the application of the standard deviation as a dispersion index. Transactional leadership had the most deviation from its mean with a standard deviation value of 0.614. This value of standard deviation suggests that there is a significantly high variation in the perception of the respondents towards transactional leadership. In other words, the respondents in the survey differ the most from each other in this variable. Conversely, hotel performance had the lowest value of deviation from the mean with a standard deviation value of 0.481.

### 4.3 Assessing structural model’s collinearity issues

Before assessing the structural model, it is crucial to address the collinearity issues in the inner structural model (predictor-criterion collinearity) to avoid misleading or bias in the regression results. The multicollinearity problem arises when two or more variables are not independent of each other This can be determined through the collinearity assessment in terms of the variance inflation factor (VIF). The common rules of thumb in assessing the potential collinearity issues are the VIF value of 5 or higher (Hair et al., 2016). Based on the collinearity assessment analysis, the VIF value for TFL = 1.995, TSL = 2.453, and CSM is 1.987, which shows that there are no potential collinearity issues in the model. Harman's single factor test was executed to check common method bias [82]. The unrotated single latent factor was 26.36% which was less than 50% [82], signifying no concern of common method variance.

### 4.4 Measurement model

The ability to verify the validity of measurements is one of the main advantages of the SEM. In this situation, construction validity applies simply to the precision of the measurements [79]. Construct validity is tested by two key instruments, convergence validity and discriminant validity in SEM analyses.

#### 4.4.1 Convergent validity relates to the similarity between the items that indicate a particular variable's degree of variance

As Table 2 shows, the factor loadings of CP7 and CC7 have the values of 0.118 and 0.157 respectively when the standardized loading of the items of the model is evaluated. Since both values are lower than the cut off point of 0.6, the respective items have been excluded from the model. As the removal of two items, in comparison to the total number of items in the constructs was not high, there was no corresponding significant change in the constructs' content as conceptualized.

**Table 2:** A representation of the results of the measurement model’s reliability and convergent validity.

Construct	Item	Factor Loading	Average Variance Extracted (AVE)^a^	Composite Reliability (CR)^b^	Internal Reliability Cronbach Alpha
Attributed (ATB)	ATB1	0.851	0.692	0.900	0.852
	ATB2	0.831			
	ATB3	0.835			
	ATB4	0.811			
Behaviour (BHV)	BHV1	0.792	0.700	0.903	0.857
	BHV2	0.831			
	BHV3	0.885			
	BHV4	0.835			
Motivation (MTV)	MTV1	0.810	0.697	0.902	0.855
	MTV2	0.852			
	MTV3	0.869			
	MTV4	0.807			
Stimulation (STM)	STM1	0.845	0.709	0.907	0.863
	STM2	0.857			
	STM3	0.822			
	STM4	0.843			
Considerations (CNS)	CNS1	0.839	0.719	0.911	0.870
	CNS2	0.875			
	CNS3	0.830			
	CNS4	0.847			
Contingent Reward (CRW)	CRW1	0.813	0.694	0.901	0.853
	CRW2	0.829			
	CRW3	0.845			
	CRW4	0.844			
Management-by-Exception-Active (MEA)	MEA1	0.844	0.735	0.917	0.880
	MEA2	0.868			
	MEA3	0.858			
	MEA4	0.858			
Management-by-Exception-Passive (MEP)	MEP1	0.848	0.706	0.905	0.861
	MEP2	0.883			
	MEP3	0.827			
	MEP4	0.800			
Signal Detection (CD)	CD1	0.847	0.715	0.926	0.900
	CD2	0.825			
	CD3	0.859			
	CD4	0.880			
	CD5	0.816			
Preparation (CP)	CP1	0.754	0.576	0.905	0.877
	CP2	0.748			
	CP3	0.745			
	CP4	0.741			
	CP5	0.767			
	CP6	0.785			
	CP7	0.118^c^			
	CP8	0.773			
Containment (CC)	CC1	0.758	0.611	0.904	0.873
	CC2	0.792			
	CC3	0.810			
	CC4	0.809			
	CC5	0.789			
	CC6	0.728			
	CC7	0.157^c^			
Recovery (CR)	CR1	0.756	0.611	0.863	0.789
	CR2	0.799			
	CR3	0.816			
	CR4	0.755			
Learning (CL)	CL1	0.724	0.607	0.860	0.783
	CL2	0.822			
	CL3	0.796			
	CL4	0.771			
Financial (FP)	FP1	0.860	0.714	0.926	0.900
	FP2	0.813			
	FP3	0.817			
	FP4	0.863			
	FP5	0.870			
Customer (CUP)	CUP1	0.774	0.636	0.875	0.809
	CUP2	0.826			
	CUP3	0.789			
	CUP4	0.800			
Internal Process (IP)	IP1	0.765	0.646	0.879	0.817
	IP2	0.792			
	IP3	0.851			
	IP4	0.804			
Learning and Growth (LG)	LG1	0.827	0.641	0.877	0.813
	LG2	0.765			
	LG3	0.818			
	LG4	0.791			
Transformational Leadership (TFL)	Attributed (ATB)	0.822	0.629	0.894	0.850
	Behaviour (BHV)	0.816			
	Motivation (MTV)	0.652			
	Stimulation (STM)	0.846			
	Considerations (CNS)	0.815			
Transactional Leadership (TSL)	Contingent Reward (CRW)	0.780	0.633	0.838	0.710
	Management-by-Exception-Active (MEA)	0.819			
	Management-by-Exception-Passive (MEP)	0.788			
Crisis Management (CSM)	Signal Detection (CD)	0.956	0.701	0.921	0.891
	Preparation (CP)	0.850			
	Containment (CC)	0.789			
	Recovery (CR)	0.733			
	Learning (CL)	0.843			
Hotel Performance (HTP)	Financial (FP)	0.809	0.649	0.881	0.820
	Customer (CUP)	0.837			
	Internal Process (IP)	0.806			
	Learning and Growth (LG)	0.772			

The modified model was further tested in order to maintain the factor structure's reliability. The result shows that the second standardized factor loading for the items and first order constructs are higher than the cut off point of 0.6 as suggested by Hair et al. (2006). The range of the values from the second assessment is 0.652–0.956.

The reliability of each construct is evaluated after the uni-dimensionality of the constructs has been attained. In order to evaluate reliability, the average variance extracted (AVE) as well as construct reliability (CR) and Cronbach’s alpha were used. The total amount of variance displayed by the indicators of the latent construct accounts for it being reflected in the average variance extracted (AVE). Table 2 shows that for all the constructs, the average variance extracted (AVE) is also greater than the cut off point of 0.5 as proposed by Nunnally & Bernstein [83]. The values of the AVE are within the range of 0.576 and 0.735. The degree to which the indicators reflect the latent concept is revealed by construct reliability (composite reliability). The values of the construct reliability for all the constructs in this study exceeded the acceptable value of 0.6 as proposed by Bagozz & Yi [84]. The values are within the range of 0.838 and 0.926.

The extent to which any measure is error-free is described by the Cronbach’s alpha value of such a measure. The values of the Cronbach’s alpha in this study are within the range of 0.710 and 0.900. This implies that all the values are also greater than the recommended value of 0.7 proposed by Nunnally & Bernstein [83] and that all constructs' Cronbach’s alpha are error-free.

#### 4.4.2 Discriminant validity

This research evaluated the discriminant validity using the HTMT Discrimination Criterion. Table 3 shows the results of HTMT discriminant criteria in evaluating the measurement model’s discriminant validity.

**Table 3:** Results of HTMT discriminant criteria.

	CSM	HTP	TFL	TSL
CSM				
HTP	0.815			
TFL	0.680	0.686		
TSL	0.863	0.735	0.881	

The HTMT values for all latent constructs were less than 0.90 as seen in Table 3. The range of the values was between 0.680 and 0.881. This confirms that there is total discrimination among the latent constructs [85].

### 4.5 Structural models

#### 4.5.1 Direct effects of hypotheses

**Fig. 2:**
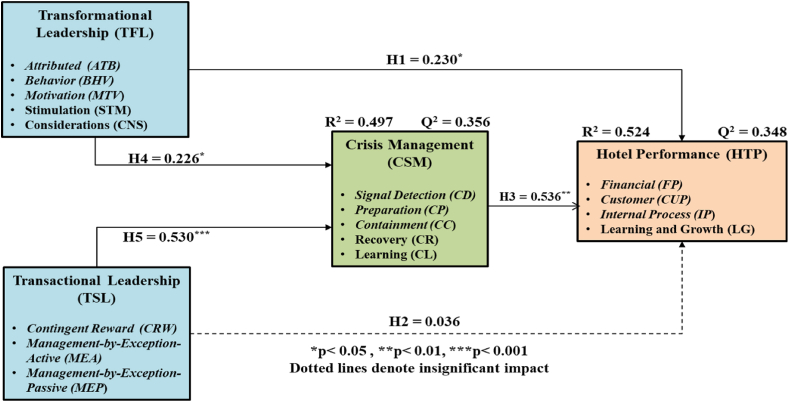
PLS analysis of the structural model for direct effects.

The direct effect of the hypotheses correlates with hypotheses H1, H2, H3, H4 and H5. A summary of the structural model utilized in testing the direct effects of hypotheses is provided in Fig. 2.

The R^2^ values were estimated at 0.497 and 0.524 for crisis management (CSM) and hotel performance (HTP). This shows that the error variance of hotel performance (HTP) is estimated to be 47.6% (100%–52.4%) of the calculated variance of hotel performance (HTP). This reveals that the three predictors can explain only 52,4% of variations in hotel performance (HTP). In the overall results, the R^2^ values were shown to meet the recommended requirement of Cohen [86] which is the 0.3 cut off value. The Q^2^ value for crisis management (CSM) together with that of hotel performance (HTP) are respectively 0.356 and 0.348. According to Chin [87], these values which are higher than zero refer to the predictive relevance of the utilized model. In summary, a high level of predictive relevance together with an acceptable fit is exhibited by the model.

As seen in Table 4, the P- values below the acceptable significance level of 0.05 statistically are significant in all paths (excluding transactional leadership on hotel performance). Therefore, H1, H3 and H4 and H5 are supported by the results. By contrast, H2 was rejected as the P-value of the hypothesis was above the acceptable level of 0.05.

**Table 4:** Results of the examination of the direct effects.

Path Shape	Path Coefficient	Standard Deviation	T-value	P-value	F-squared	Effect Size	Result
TFL → HTP	0.230*	0.106	2.169	0.030	0.056	Small	H1) Supported
TSL → HTP	0.036	0.103	0.348	0.728	0.001	No effect size	H2) Rejected
CSM → HTP	0.536**	0.158	3.381	0.001	0.303	Medium	H3) Supported
TFL → CSM	0.226*	0.097	2.336	0.020	0.054	Small	H4) Supported
TSL → CSM	0.530***	0.066	8.014	0.000	0.295	Medium	H5) Supported

*p < 0.05, **p < 0.01, ***p < 0.001.

#### 4.5.2 Mediation effect of constructs

In testing for mediation, the SEM technique is preferred over regression techniques because SEM permits both measurements, as well as structural relationships, to be modelled in addition to yielding overall values of fit indices [88]. Table 5 presents the mediating role of crisis management on transformational leadership and transactional leadership relationship and hotel performance. Table 5 indicates that both Hypothesis 6 and Hypothesis 7 were supported.

**Table 5:** Results of examination of the mediation effects.

DV = Hotel Performance M = Crisis Management	Independent Variables	
	Transformational Leadership	Transactional Leadership
Total Effect of IV on DV without M (path a)	0.351*** ^(sig:0.000)^	0.320*** ^(sig:0.000)^
Direct Effect of IV on DV with M (path a’)	0.230* ^(sig:0.030)^	0.036 ^(sig:0.728)^
Indirect Effect of IV on DV through M (path bc)	0.121* ^(sig:0.050)^	0.284**^(sig:0.001)^
Effect of IV on M (path b)	0.226*^(sig:0.020)^	0.530*** ^(sig:0.000)^
Effect of M on DV (path c)	0.536*** ^(sig:0.001)^	0.536*** ^(sig:0.001)^
Mediation Path	TFL→CSM→HTP	TSL→CSM→HTP
Mediation Effect	Yes	Yes
Degree of Mediation	Partial	Full
Hypothesis Result	H6) Supported	H7) Supported

*p < 0.05, **p < 0.01, ***p < 0.001.

#### 4.5.3 Moderation effects of leader’s experience (LDE)

According to Hair et al. [79], the effect of an independent variable on a dependent variable is based on another variable’s value that moderates the relationship - then the moderation effect takes place. As seen in Table 6, the moderating effects of leader’s experience were examined. In addition, the path coefficient was used for evaluating the contribution of each interaction term to the dependent variable.

**Fig. 3:**
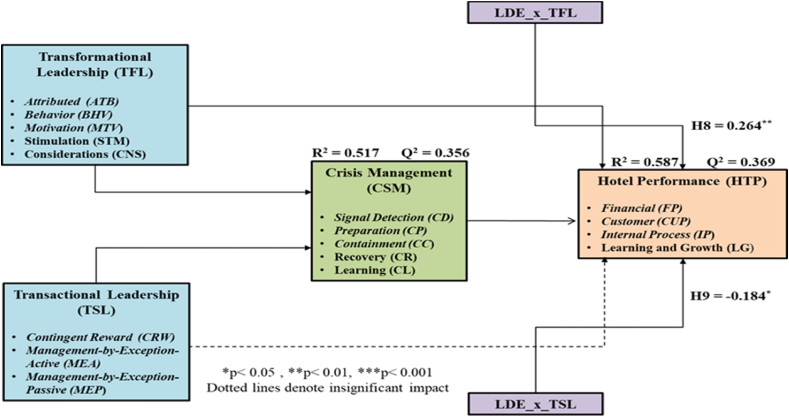
PLS analysis of the structural model for moderation effects of leader’s experience.

Fig. 3 shows the structural model with its interaction terms used in examining the moderating effects of leader’s experience.

The respective value of R-squared (R^2^) for hotel performance (HTP) is 0.587. This value is higher than the recommended threshold of 0.3 [86]. The values of Q-squared (Q^2^) for hotel performance (HTP) was 0.369. This value was far above 0 and hence represents the model’s predictive relevance [87]. Therefore, a high level of predictive relevance, together with an acceptable fit, was exhibited by the model.

As seen in Table 6, there were significant effects on Hotel Performance (HTP) and the relationship of leader’s experience (LDE) with transformational leadership (TFL). This was also true for the interaction terms of leader’s experience (LDE) and transactional leadership (TSL). The P-value for both interaction terms were below the acceptable significant threshold value of 0.05. These results show that leadership experience (LDE) moderates the effects that transformational leadership (TFL) and transactional leadership (TSL) have on hotel performance (HTP). Therefore, hypothesis H8 and hypothesis H9 are supported.

**Table 6:** Results of the moderation effects of leader’s experience.

Path Shape	Path Coefficient	Standard Deviation	T-value	P-value	F-squared	Effect Size	Result
LDE*TFL→HTP	0.264**	0.100	2.648	0.008	0.076	Small	H8) Supported
LDE*TSL→HTP	−0.184*	0.073	2.541	0.011	0.042	Small	H9) Supported

*p < 0.05, **p < 0.01, ***p < 0.001.

## 5 Discussion

This study examined the relationship between leadership styles, leaders' experience, crisis management and hotel performance from the perspectives of Contingency Theory, Transactional-transformational Leadership Theory and the Resource-based View Theory. Based on a questionnaire survey of 119 respondents currently holding managerial positions in Jordanian 3 to 5-star hotels, the Partial Least Square (PLS) Structural Equation Modelling (SEM) analysis showed the results from examining the direct effect hypothesis that the transformational leadership style has significant positive effects on hotel performance. The result is consistent with research by Sobaih et al. [17], Zumitzavan & Udchachone [24], and Cheung, King & Wong [26] that demonstrates the considerable impact of transformative leadership on hotel performance. Regarding the impact of transformational leadership on crisis management, the findings are consistent with those of a prior study [[49], [50], [51]] that demonstrated this impact to be positive and significant.

Transactional leadership, on the other hand, has an insignificant effect on hotel performance, according to the study. The findings are consistent with those of Radwan’s [32] study on transactional leadership and Egyptian hotel performance. Yet, it has been discovered that transactional leadership has a favourable, significant impact on crisis management, which is consistent with the findings of a prior study by Heuvel [57] and Zohar & Luria [58]. The findings demonstrate that, from the perspective of Jordan’s hotel industry, transformational leadership has a greater impact on crisis management practises rather than transactional leadership.

In terms of the mediating effect, it has been discovered that the relationship between transformational leadership and hotel performance was partially mediated by crisis management. The outcome demonstrates that high levels of transformational leadership result in a stronger adoption of crisis management, which improves hotel performance. The relationship between transactional leadership and hotel performance, on the other hand, has been found to be fully mediated by crisis management, which shows that crisis management can account for the relationship between transactional leadership and hotel performance. This study’s findings are consistent with those of studies by Zhao et al. [59] and Nitzl et al. [60]. This argument is supported by Zhao, Lynch Jr & Chen [59], as well as Nitzl, Roldan & Cepeda [60].

Moreover, it was found that leader experience had a positive moderating effect on the relationship between transformational leadership style and hotel performance, while the relationship between transactional leadership style and hotel performance has a negative moderating effect. The results of the study are consistent with the Contingency Theory which argues that leadership style is contingent upon external and internal factors [62], and the Resource-based View Theory which argues that leadership experience is one of the most valuable and unique resources for a hotel when facing a turbulent business environment [91].

Although previous research has demonstrated that crisis management has a major effect on hotel performance, such as Majli and Tamimi [40], Armoo [41], and Laba [42], the importance of crisis management practices in linking the leader with various leadership styles and sustaining hotel performance impacted by the crisis is demonstrated by the mediating role discovered in this study. According to the findings of this study, transactional leadership styles have a statistically greater impact on crisis management practices than transformational leadership styles. However, the impact of crisis management practices on sustainability performance with inclusion of leadership styles used in the study is strongest with transactional leadership styles. In terms of leader experience, it can provide a transformational leader with energy for rapid problem solving and execution under high-stress and chaotic conditions by organising a network of effective teams. Effective teams led by experienced leaders can speed up external communication processes, financial stress testing, supply chain processes, communication across employee channels, and the hotel’s asset management. Experienced leaders also can promote psychological safety to their employees, allowing them to openly discuss ideas, questions and concerns without fear of repercussions.

Based on the descriptive statistics of the study, the mean of the variables used in this study, such as transformational leadership (mean = 3.024), transactional leadership (mean = 3.028), crisis management (mean = 3.002), and hotel performance (mean = 3004), has shown a moderate level. The highest mean is represented by the transactional leadership variables. In contrast, the lowest mean represents the crisis management variable. It indicates that there is still room for improvement for the hotel’s owner/manager to improve their leadership styles, crisis management, and hotel performance. The hotel’s owner/managers should be exposed to ongoing training related to crisis leadership by the various experts in the hotel industry to ensure that they are well equipped to face the prolonged crisis faced by the hotel industry.

## 6 Conclusion

The current environment of volatility, uncertainty, complexity and ambiguity (VUCA) has continued to contribute to unpredictability in the hotel industry. Leadership in crisis management is one of the crucial assets for the hotel industry. The conclusions drawn from the findings are the following: transformational leadership and crisis management have a direct positive effect on hotel performance which is significant. Hence, these variables should be directly manipulated and harnessed to achieve enhanced hotel performance. Transactional leadership does not directly influence hotel performance, thus suggesting the probable role of crisis management as mediator. Moreover, transformational leadership, as well as transactional leadership, have a direct, positive effect on crisis management which means these leadership styles, when effectively utilized, are vital in implementing crisis management strategies in the midst of crisis. Additionally, it was found that crisis management, followed by transformational leadership, were the most important factors for hotel performance; transactional leadership was found to be the most important element of crisis management. In addition, crisis management plays the role of a complementary/partial mediator in the relationship between transformational leadership and hotel performance while crisis management fully mediates the relationship between transactional leadership and hotel performance. Furthermore, on the one hand, it is suggested that a leader’s experience positively moderates the relationship between transformational leadership and hotel performance. This indicates that an increase in the level of the leader’s experience as moderator results in a corresponding positive enhancement of the effect of transformational leadership on hotel performance. On the other hand, the leader’s experience negatively moderates the relationship between transactional leadership and hotel performance.

The findings show that the establishment of interventions for crisis management act as a significant mediator in the ability of a hotel leader to make essential changes relevant to the situation. Using the right skills for the right conditions in the midst of crisis, thus mitigate any negative impact on hotel performance. This demonstrates that Jordan’s hotel sector leaders should make crisis management practices a continuous process in order to respond to challenges and prevent future crises. Crisis management has been identified as an effective tool that supplements traditional leadership styles in improving hotel sustainability during a crisis. The findings show that crisis management is compatible with different types of leadership styles, although we can see that the transactional leader has a stronger effect than a leader with a transformational leadership style. The study’s conceptual framework contributes to the literature in the field of transformational leadership and transactional leadership as an independent variable, crisis management as a mediating variable, leader’s experience as a moderating variable and hotel performance as a dependent variable in the context of the Jordanian and Middle Eastern hotel sectors. The findings highlight the role of both leadership types and crisis management in enhancing hotel performance in Jordanian hotels.

## 7 Implications of the research findings, limitations and areas for future research

This study makes a significant contribution to the scientific body of knowledge and contributes theoretically by incorporating transformational and transactional leadership, crisis management and hotel performance in one model. This research could enable the creation of a much-needed foundation for future impactful studies in the domain of leadership and crisis management, as well as answer pertinent questions that future researchers can build upon. Furthermore, crisis management can be used to clarify the association between leadership styles and performance of hotels in the hotel sector and perhaps be extended to the entire tourism industry. Also, the literature reveals that researchers have largely ignored crisis management studies in empirical research in particular [89,90]. While this study aims to extend existing research into crisis management, it more significantly fills a gap in the literature. Although current leadership investigations have leaned towards a focus on the influence of leadership styles in normal times, very little consideration has been paid to its influence in crisis situations in places such as Jordan and other Middle Eastern countries in present times.

Leadership effectiveness has been considered a most important issue for several organisations, both during normal times and in times of crisis, hence this study is a very significant addition to leadership literature due to the fact that leadership in crisis situations has been empirically studied. The findings of this study also prove beneficial to Contingency Theory and the Resource-based View Theory by addressing the existing void in the literature in relation to the application of Contingency Theory to effective leadership styles in a crisis situation while leveraging on organisations' resources to develop a competitive advantage in business [91]. By applying the principles of Contingency Theory, Transactional and Transformational Leadership Theory and the resource-based view and expanding them to a broader base with contextual modifications, a foundation is laid for other researchers to apply this knowledge in future research into the application of crisis management strategies for optimum performance. This present study offers vital practical insights with regards to the intervention of crisis management on leadership styles to enhance hotel performance. The findings are significant for hotel managers who could benefit from the findings in several ways.

Firstly, the crisis measurement indicators consisting of 5 phases used in this present study (signal detection, preparation, containment, recovery and learning) are a valuable source of knowledge for hotel managers. It helps them to identify important areas where improvements are needed in their crisis management framework, as well as providing a pathway for hotel practitioners/owners to invest in necessary capabilities to enhance their crisis management strategies. Secondly, the findings can benefit other tourist sectors in Jordan by providing a base for further studies into leadership styles and the intermediary role of crisis management on performance. This can effectively and efficiently facilitate crisis management operations in other sectors such as the food and beverage industry, airline industry etc. Thirdly, managers are recommended to adopt a leadership style which ensures that existing resources will effectively be incorporated and maximized within the internal and external environment in order to meet organisational and environmental challenges. Also, in view of the results pertaining to a leader’s experience in this study, it is important for hotel owners and human resource executives to proactively recruit and develop managers with significant experience to handle crisis events. They should also appoint managers for crisis management planning in the organisation. A significant criteria for this appointment should be the years of experience of the appointee. This is because this study, which supports the findings of earlier studies [49,70,72], shows that leaders with prior experience are more capable of ensuring sustained performance.

Despite its importance, the study has several limitations. This study’s cross-sectional design may limit the researcher’s interpretation of the dynamism of crisis management practices and leadership styles over time. As a result, it is strongly advised that any future studies employ longitudinal analysis. Furthermore, the current study relied solely on subjective responses when gathering hotel data on crisis management and hotel performance, based on an adapted questionnaire survey instrument. This could be improved by supplementing subjective input as needed with objective company data from secondary sources. A future study could also look into contemporary leadership styles, such as autocratic, democratic, directive, supportive, affiliative, coaching, servant and laissez-faire, and how they affect crisis management practices.

## Author contribution statement

Amar Hisham Jaaffar: Conceived and designed the experiments; Contributed reagents, materials, analysis tools or data; Wrote the paper.

Raed Hussam Alzoubi: Performed the experiments; Analyzed and interpreted the data; Contributed reagents, materials, analysis tools or data; Wrote the paper.

Alkharabsheh Omar Hamdan Mohammad: Analyzed and interpreted the data; Contributed reagents, materials, analysis tools or data.

Jegatheesan Rajadurai: Conceived and designed the experiments; Performed the experiments.

## Data availability statement

The data that has been used is confidential.

## Declaration of competing interest

The authors declare that they have no known competing financial interests or personal relationships that could have appeared to influence the work reported in this paper.
