# Supplementary material for: Leadership and crisis management and their link to improvement of hotel performance: A study of the Jordanian hotel sector
Source: Heliyon. 2023 Jul 2;9(7):e17839. doi: 10.1016/j.heliyon.2023.e17839 (PMC10395140; doi:10.1016/j.heliyon.2023.e17839)
Supplement: Multimedia component 1 [file mmc1.docx]

Survey Questionnaire (English Version)

**SURVEY QUESTIONNAIRE**

**Attention: Hotel Owner/ General Manager/ Deputy General Manager/ Department Manager (Please do not answer this questionnaire, if you have been working in this hotel less than three years).**

**SECTION A: DEMOGRAPHIC PROFILE**

**Please** **tick (**√) **the appropriate box**

**PART 1: BACKGROUND**

1. Please indicate your gender: Male Female

2. Please indicate your age group:

Less than 25 years 25-35 36-45 46-55 56-64 Over 64

3. Your current position:

Hotel Owner General Manager Deputy General Manager Department Manager

Other (please specify): ……………………………………………………………………...

4. How many years have you been working in your current position in the hotel sector?

Less than 3 years 3–6 years 7-10 years 11-14 years More than 14 years

**PART 2: HOTEL PROFILE**

1. Star rating:

5 4 3

2. Type of your Hotel's affiliation:

Chain Independent Other (please specify): ………...

3. Region of the hotel: North South Middle

4. How many years has your hotel been in operation?

Less than 3 years 3-6 years 7-10 years 11-14 years More than 14 years

5. Number of Rooms:

50 and below 51-100 101-150 151-200 201 or more

6. Number of employees:

50 and below 51-100 101-150 151-200 201 and above

**SECTION B: TRANSFORMATIONAL LEADERSHIP**

| In this section, the researcher is interested in your opinions about the **Transformational leadership** dimensions in your hotel.  **Transformational leadership** is the extent to which the behaviour/attitude of a leader can instigate an increase in the abilities, aspirations and motivation levels of his/her followers who will sacrifice their self-interests for the achievement of the objectives of an organisation.  **Please mark with " x" one answer that best represents your experiences and opinions for the following statements.**  **1= Not at All 2= Once in a While 3= Sometimes 4= Fairly Often 5= Frequently, If Not Always** |
| --- |

| **5** | **4** | **3** | **2** | **1** | **Survey Statement** | **Index** | |
| --- | --- | --- | --- | --- | --- | --- | --- |
| **Idealized influence (attributed)** | | | | | | | **Transformational leadership** |
|  |  |  |  |  | I instil pride in others for being associated with me. | **ATB.1** |  |
|  |  |  |  |  | I go beyond self-interest for the greater good of the group. | **ATB.2** |  |
|  |  |  |  |  | I act in ways that build others’ respect for me. | **ATB.3** |  |
|  |  |  |  |  | I emphasize the importance of self-confidence. | **ATB.4** |  |
| **Idealized influence (behaviour)** | | | | | | |  |
|  |  |  |  |  | I talk about my most important values. | **BHV.1** |  |
|  |  |  |  |  | I specify the importance of having a strong sense of purpose. | **BHV.2** |  |
|  |  |  |  |  | I pay attention to my decision’s outcomes. | **BHV.3** |  |
|  |  |  |  |  | I emphasize the importance of having a collective sense of mission. | **BHV.4** |  |
| **Inspirational motivation** | | | | | | |  |
|  |  |  |  |  | I speak with optimism about the future. | **MTV.1** |  |
|  |  |  |  |  | I talk enthusiastically about what needs to be accomplished. | **MTV.2** |  |
|  |  |  |  |  | I articulate a compelling vision of the future. | **MTV.3** |  |
|  |  |  |  |  | I show determination to achieve the goals of my organisation. | **MTV.4** |  |
| **Intellectual stimulation** | | | | | | |  |
|  |  |  |  |  | I re-examine appropriateness about critical business assumptions. | **STM.1** |  |
|  |  |  |  |  | I seek differing perspectives when solving problems. | **STM.2** |  |
|  |  |  |  |  | I consider various opinions from others. | **STM.3** |  |
|  |  |  |  |  | I suggest new ways of looking at how to complete assignments. | **STM.4** |  |
| **Individualized considerations** | | | | | | |  |
|  |  |  |  |  | I take time to instruct others. | **CNS.1** |  |
|  |  |  |  |  | I treat others as individuals rather than just a member of a group. | **CNS.2** |  |
|  |  |  |  |  | I consider an individual as having different needs, abilities, and aspirations different from others. | **CNS.3** |  |
|  |  |  |  |  | I help others to develop their strength. | **CNS.4** |  |

**SECTION C: TRANSACTIONAL LEADERSHIP**

| In this section, the researcher is interested in your opinions about the **Transactional leadership** dimensions in your hotel.  **Transactional leadership** is the use of rewards as well as promises of rewards by the leaders to instigate improvement in their followers’/employees’ performance.  **Please mark with " x" one answer that best represents your experiences and opinions for the following statements.**  **1= Not at All 2= Once in a While 3= Sometimes 4= Fairly Often 5= Frequently, If Not Always** |
| --- |

| **5** | **4** | **3** | **2** | **1** | **Survey Statement** | **Index** | |
| --- | --- | --- | --- | --- | --- | --- | --- |
| **Contingent reward** | | | | | | | **Transactional leadership** |
|  |  |  |  |  | I help others overcome their disabilities/shortcomings. | **CRW.1** |  |
|  |  |  |  |  | I assign roles and responsibilities to optimise performance. | **CRW.2** |  |
|  |  |  |  |  | I make clear what one can expect to receive when performance goals are achieved. | **CRW.3** |  |
|  |  |  |  |  | I clarify incentive regulations. | **CRW.4** |  |
| **Management-by-exception-active** | | | | | | |  |
|  |  |  |  |  | I focus attention on irregularities, mistakes, exceptions, and deviations from standards. | **MEA.1** |  |
|  |  |  |  |  | I concentrate my full attention on dealing with mistakes, complaints, and failures. | **MEA.2** |  |
|  |  |  |  |  | I track others’ mistakes. | **MEA.3** |  |
|  |  |  |  |  | I direct my attention toward failures to meet standards. | **MEA.4** |  |
| **Management-by-exception-passive** | | | | | | |  |
|  |  |  |  |  | I fail to interfere until problems become serious. | **MEP.1** |  |
|  |  |  |  |  | I wait for things to go wrong before taking action. | **MEP.2** |  |
|  |  |  |  |  | I show that I am a firm believer in “if it ain’t broke, don’t fix it”. | **MEP.3** |  |
|  |  |  |  |  | I demonstrate that problems must become chronic before I take action. | **MEP.4** |  |

**SECTION D: CRISIS MANAGEMENT**

| In this section, the researcher is interested in your opinions about the **Crisis management** dimensions in your hotel.  **Crisis management** can be defined as an ongoing integrated and comprehensive effort that organisations effectively put into place in an attempt to first and foremost understand and prevent a crisis, and to effectively manage those that occur, taking into account, at each and every step of their planning and training activities, the interest of their stakeholders.  **Please mark with " x" one answer that best represents your experiences and opinions for the following statements.**  **1= Strongly Disagree 2= Disagree 3= Neutral 4= Agree 5= Strongly Agree** |
| --- |

| **5** | **4** | **3** | **2** | **1** | **Survey Statement** | **Index** | |
| --- | --- | --- | --- | --- | --- | --- | --- |
| **Crisis signal detection** | | | | | | | **Crisis management** |
|  |  |  |  |  | There is a special unit in our hotel to observe crises indicators. | **CD.1** |  |
|  |  |  |  |  | The top management is focused to collect and detect the signs of risks which are expected to be the crises indicators. | **CD.2** |  |
|  |  |  |  |  | There are interests in the process of classifying and analysing crises indicators. | **CD.3** |  |
|  |  |  |  |  | There are qualified crews working on rating and analysing crises indicators. | **CD.4** |  |
|  |  |  |  |  | The internal and external workplace has been surveyed comprehensively and systematically to recognize expected crises indicators. | **CD.5** |  |
| **Crisis preparation** | | | | | | |  |
|  |  |  |  |  | The hotel's organisational chart is complete and flexible to allow usefulness when dealing with occurred crises. | **CP.1** |  |
|  |  |  |  |  | An effective group has been designed to solve several expected crises. | **CP.2** |  |
|  |  |  |  |  | Appropriate support is provided to assist responsible party to plan in diagnosing the expected crises. | **CP.3** |  |
|  |  |  |  |  | Clear management briefing has specified the process of dealing with the expected crises. | **CP.4** |  |
|  |  |  |  |  | We provide possible resources required (materials, human, technology, information) in preparing to face the expected crises. | **CP.5** |  |
|  |  |  |  |  | Cooperative effort is available between institutions and government related agencies in crises’ management area. | **CP.6** |  |
|  |  |  |  |  | There are scheduled meetings to perform mock test in dealing with the expected crises. | **CP.7** |  |
| **Crisis containment** | | | | | | |  |
|  |  |  |  |  | We have successfully control crises when arise and refrain it from spreading continuously in a timely manner. | **CC.1** |  |
|  |  |  |  |  | Fast action taken has resulted in reducing the harmful effects of the crisis. | **CC.2** |  |
|  |  |  |  |  | Time management is a crucial factor being taken into account when dealing with crises. | **CC.3** |  |
|  |  |  |  |  | There is adequate capacity and fast action to mobilize the necessary material and human resources to contain the crisis. | **CC.4** |  |
|  |  |  |  |  | Effective communication medium is available to detect factors effecting crises. | **CC.5** |  |
|  |  |  |  |  | Top management responds to the distribution of tasks and powers over a short and appropriate period at the crisis time. | **CC.6** |  |
| **Crisis recovery** | | | | | | |  |
|  |  |  |  |  | Even when dealing in crises period, top management has taken appropriate action to ensure daily operational tasks is performed in a timely manner as per usual practice. | **CR.1** |  |
|  |  |  |  |  | Top management is responsible to reduce the harmful effects of crises as well as stopping the crises from continuously occurring. | **CR.2** |  |
|  |  |  |  |  | In order to reduce crisis effects, top management has provided assistance to departments affected by crisis. | **CR.3** |  |
|  |  |  |  |  | Top management has provided sufficient support and authority to managers to solve crises. | **CR.4** |  |
| **Crisis learning** | | | | | | |  |
|  |  |  |  |  | Benefits to future are expected based on lesson learnt from previous crises faced by top management. | **CL.1** |  |
|  |  |  |  |  | Top management is responsible to evaluate crises management and plan for future improvement. | **CL.2** |  |
|  |  |  |  |  | Top management is responsible to ensure lesson learnt from previous experience being collected for future benefits. | **CL.3** |  |
|  |  |  |  |  | Top management is responsible to learn from other agencies on method to deal with crises. | **CL.4** |  |

**SECTION E: HOTEL PERFORMANCE**

| In this section, the researcher is interested in your opinions about the **Hotel performance** dimensions in your hotel.  **Hotel performance** is concerned with the final outcomes of hotel operation activities. It has four dimensions: financial, customer, internal process, and learning and growth; these must be combined in order to provide a comprehensive view of organisational performance.  **If your hotel is less than three years in operation, please don't answer this part.**  **Please mark with "x" one answer that indicates the changes in performance of your hotel over the past 3 years.**  **1= Strongly Disagree 2= Disagree 3= Neutral 4= Agree 5= Strongly Agree** |
| --- |

| **5** | **4** | **3** | **2** | **1** | **Survey Statement** | **Index** | |
| --- | --- | --- | --- | --- | --- | --- | --- |
| **Financial perspective** | | | | | | | **Hotel performance** |
|  |  |  |  |  | The total running costs of the hotel has decreased. | **FP.1** |  |
|  |  |  |  |  | The unexpected losses in the hotel have been reduced. | **FP.2** |  |
|  |  |  |  |  | The rate of sales growth has increased. | **FP.3** |  |
|  |  |  |  |  | The worth of hotel assets has increased. | **FP.4** |  |
|  |  |  |  |  | The profit of the hotel has increased. | **FP.5** |  |
| **Customer perspective** | | | | | | |  |
|  |  |  |  |  | The needs of various types of customers have been satisfied. | **CUP.1** |  |
|  |  |  |  |  | Customer loyalty has increased. | **CUP.2** |  |
|  |  |  |  |  | Customer’s satisfaction has increased. | **CUP.3** |  |
|  |  |  |  |  | Compared to our competitors, the number of our customers have increased. | **CUP.4** |  |
| **Internal process perspective** | | | | | | |  |
|  |  |  |  |  | The operating efficiency of hotel has increased. | **IP.1** |  |
|  |  |  |  |  | Customer complaints have decreased. | **IP.2** |  |
|  |  |  |  |  | The ability to retain old customers has improved. | **IP.3** |  |
|  |  |  |  |  | We have improved in our ability to target our customers. | **IP.4** |  |
| **Learning and growth perspective** | | | | | | |  |
|  |  |  |  |  | Employees’ ability to solve problems has improved. | **LG.1** |  |
|  |  |  |  |  | The quality of employees' service has improved. | **LG.2** |  |
|  |  |  |  |  | The intention of employees to learn has increased. | **LG.3** |  |
|  |  |  |  |  | The hotel culture has been promoted effectively. | **LG.4** |  |

***Thanks for your time***
